# Acupuncture plus rehabilitation for post-stroke depression: A protocol for systematic review and meta-analysis

**DOI:** 10.1097/MD.0000000000021078

**Published:** 2020-07-10

**Authors:** Yonghui Hou, Ning Zhang, Jieming Hao, Xiangzhi Wang, Zehuai Wen, Ding Luo, Baile Ning, Wenbin Fu, Ying Liu

**Affiliations:** a The First Hospital of Shijiazhuang City, Shijiazhuang, Hebei; b Shijiazhuang Community Health Service Management Center, Shijiazhuang, Hebei; c The Second Affiliated Hospital of Guangzhou University of Chinese Medicine, Guangzhou, Guangdong; d National Center for Design Measurement and Evaluation in Clinical Research, Guangzhou University of Chinese Medicine; e Situ Ling Studio of Lingnan Acupuncture School; f Famous Doctor's Studio of Academician Shi Xuemin, Guangzhou; g Shenzhen Bao’an Research Center for Acupuncture and Moxibustion, Shenzhen, Guangdong, China .

**Keywords:** acupuncture, rehabilitation, post-stroke depression, meta-analysis, protocol

## Abstract

**Background:**

Post-stroke depression (PSD) is a common stroke complication that is characterized by hopelessness, anxiety, disordered sleep, and lowered responsiveness. Rehabilitation and acupuncture treatments are often combined to treat PSD; however, there has been no meta-analysis on their synergistic effect. Therefore, we aim to perform a systematic review and meta-analysis to estimate the effectiveness of acupuncture and rehabilitation in PSD treatment.

**Methods:**

We will search the following electronic databases: PubMed, the Cochrane Library, EMBASE, the China National Knowledge Infrastructure, the Chinese Biomedical Literature Database, China Science and Technology Journal Database, and Wan Fang databases. We will include studies published between the database initiation and May 2020. Two reviewers will separately conduct study selection, data extraction, and risk of bias assessment. Disputes will be settled by consulting a third reviewer. Review Manager Software 5.3 will be employed for this meta-analysis.

**Results:**

This systematic review will assess whether acupuncture combined with rehabilitation treatment is more effective than rehabilitation alone in the management of PSD.

**Conclusion:**

This systematic review will provide evidence regarding the synergistic effect of acupuncture and rehabilitation treatment for PSD.

## 1 Introduction

### 1.1 Description of the condition

Post-stroke depression (PSD) is a common stroke complication characterized by hopelessness, anxiety, disordered sleep, and lowered responsiveness.^[1]^ In the initial 2 post-stroke months, about 36% of the patients develop signs of depression with 14% being diagnosed with major depressive disorder.^[2]^ In the People's Republic of China, 7.5 million stroke survivors have been reported; moreover, there are 3 million patients with PSD.^[3]^ PSD has harmful motor and cognitive effects and negatively affects functional recovery.^[4]^ A previous study reported that depression is an independent risk factor for stroke rehabilitation.^[3]^ Further, patients with depression have thrice the mortality in those without depression.^[5]^ Therefore, it is important to determine the appropriate PSD treatment for lowering the associated mortality and disability rate.

### 1.2 Description of the intervention

The first-choice antidepressant treatments for patients with a PSD diagnosis are well established.^[6]^ The most frequently studied agents are antidepressants; among them, selective serotonin reuptake inhibitors and serotonin and norepinephrine reuptake inhibitors are the most common.^[7]^ However, antidepressants are associated with several adverse side effects, including mouth dryness, fatigue, drowsiness, weight gain, and sexual dysfunction. This, in turn, leads to low patient compliance rates.^[8,9]^

Acupuncture is a promising effective therapy that is increasingly being globally accepted as a therapeutic option for managing various health conditions.^[10]^ Several systematic reviews and meta-analysis have suggested that acupuncture may be superior to antidepressants with respect to clinical effectiveness and the alleviation of depressive symptoms in patients with PSD.^[6,7,11]^ A review of studies included in these systematic analyses shows that acupuncture combined with rehabilitation training has been used to treat PSD.

Rehabilitation involves physical and occupational therapy. Physical therapy promotes limb function recovery and disability function compensation. Occupational treatment improves patients’ self-care ability. Moreover, improved exercise and self-care ability enhances their independence and ameliorates their depressive symptoms.^[12]^ Therefore, the use of acupuncture and rehabilitation training in PSD therapy requires further research.

### 1.3 How the intervention might work

The mechanism underlying the treatment effect of acupuncture on PSD remains unclear. The use of combined acupuncture and rehabilitation therapy might reduce the severity of depression through positive effects on the prognosis of post-stroke neurological symptoms (limb disorders, dysphagia, aphasia, and incontinence).

### 1.4 Why is it essential to perform this review?

Combined rehabilitation treatment and acupuncture are often used for PSD treatment; however, their synergistic treatment effect has not been assessed in a meta-analysis. To evaluate this synergy based on the current literature, a systematic review could yield the highest level of evidence and allows the evaluation of the effectiveness and safety of each therapy. Therefore, this study is necessary.

### 1.5 Objectives

This meta-analysis aims to assess the effectiveness of acupuncture combined with rehabilitation in treating PSD, which could allow may provide alternative non-drug treatment options for PSD.

## 2 Methods

### 2.1 Study registration

This study protocol was prospectively registered in the International Prospective Register of Systematic Reviews (Identification number, CRD42020175205) on April 28, 2020. This protocol abides by the statement guidelines of preferred reporting items for systematic reviews and meta-analyses protocols.^[13]^

### 2.2 Inclusion criteria

#### 2.2.1 Study type

This review will include randomized controlled trials (RCTs) on acupuncture for PSD published in Chinese and English. We will exclude non-RCTs, review studies, case reports, and animal experiments.

#### 2.2.2 Participants

We will consider patients with a clinical diagnosis of PSD irrespective of their gender, age, severity, and disease duration.

#### 2.2.3 Type of intervention

The experimental group will comprise of individuals who received individual treatment with filiform needle acupuncture together with rehabilitation treatment. Studies assessing combined filiform needle acupuncture treatments with rehabilitation should use the same rehabilitation treatment protocol for the control and experimental groups. The control group should receive selective serotonin reuptake inhibitors combined with rehabilitation treatment.

#### 2.2.4 Type of outcome measures

##### 2.2.4.1 Primary outcomes

The primary outcomes will include the Hamilton depression scale score and the effective rate. As previously reported, a ≥ 25% reduction in the Hamilton depression scale score was indicative of effective treatment.^[14]^

The secondary outcomes were the National Institute of Health Stroke Scale scores, Barthel index, and the incidence of adverse events.

### 2.3 Search strategy

The following electronic databases will be searched: PubMed, the Cochrane Library, EMBASE, China National Knowledge Infrastructure, Chinese Biomedical Literature Database, China Science and Technology Journal Database, and Wan Fang databases. We will consider articles published between the database initiation and May 2020. Table 1 presents the details of the search strategy for PubMed. Similar search strategies will be used for all electronic databases.

**Table 1:**
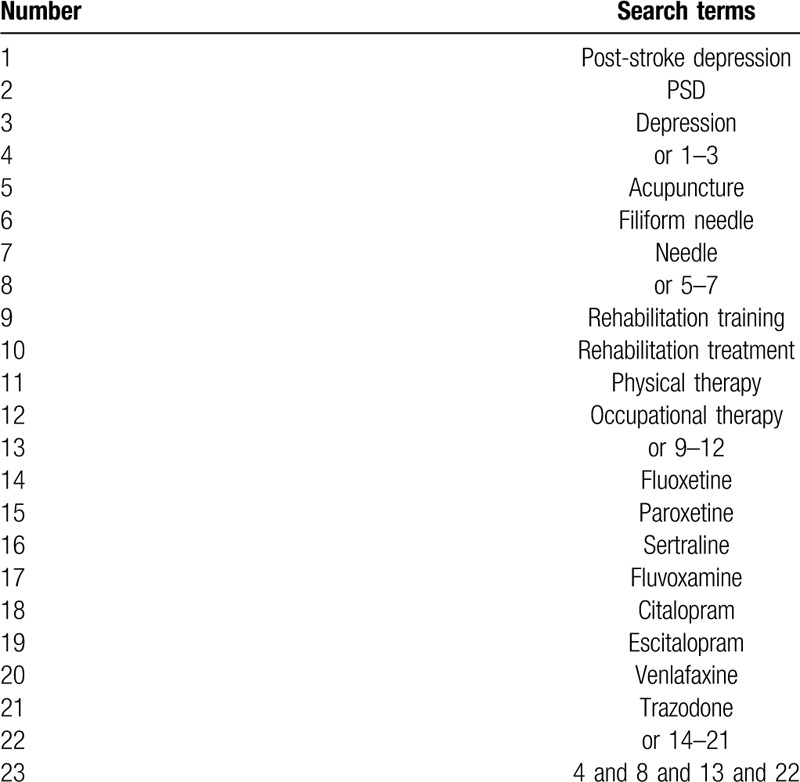
Search strategy for the PubMed database.

### 2.4 Study selection

**Figure 1:**
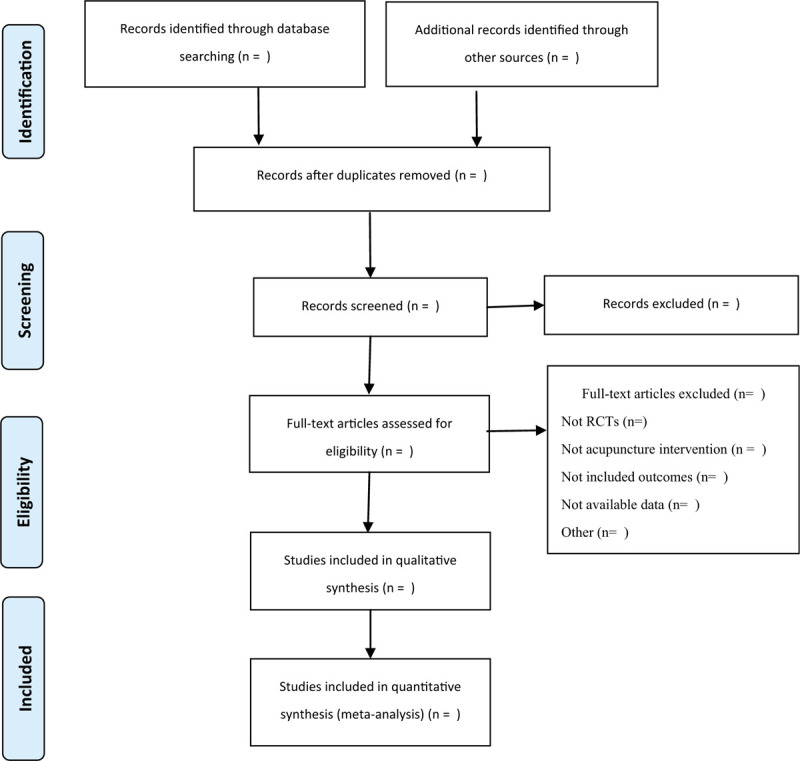
PRISMA flow chart.

One reviewer will search for potentially related studies. Subsequently, the retrieved studies will be imported into NoteExpress for duplicate counting and removal. Next, 2 reviewers will separately critique all the eligible studies. Titles and abstracts will be scanned to exclude irrelevant records. Subsequently, 2 reviewers will screen the full text for further filtration where disputes will be resolved via consultation with the third reviewer. The study selection process will be summarized as a PRISMA flowchart (Fig. 1).

### 2.5 Data extraction and management

The data will be independently extracted by 2 reviewers using a uniform data form. We will extract the following information: the journal title, first author, year of publication, study design, patient characteristics, control intervention, experimental intervention, outcomes, duration of intervention, etc. In case a study has unclear or inadequate information, we will attempt to contact the authors via email. Any dispute will be settled by consulting a third reviewer.

### 2.6 Risk of bias assessment

Two reviewers will separately assess the risk of bias of the selected RCTs using the Cochrane risk of bias assessment tool. This tool has the following 7 domains: random sequence generation, allocation concealment, blinding of participants and personnel, blinding of outcome assessment, incomplete outcome data, selective reporting, and other bias. A bias value of “low,” “unclear,” or “high” will be used to rank the risk of bias. These seven domains will be separately appraised by 2 reviews and discrepancies will be addressed by consulting a third reviewer.

### 2.7 Data synthesis and analysis

#### 2.7.1 Measurement of the treatment effect

We will use risk ratios (RR) and 95% confidence intervals (95% CI) for dichotomous variables and mean differences and 95% confidence intervals for continuous variables.

#### 2.7.2 Heterogeneity assessment

We will appraise among-study heterogeneity according to the guidelines of Review Manager 5.3.5 Software. The chi-squared test will be used for heterogeneity calculations. The random-effect and mixed-effect models will be adopted if I^2^ value ≥ 50% (significant heterogeneity) and < 50% (minor heterogeneity), respectively.

#### 2.7.3 Data synthesis

If studies are adequately homogeneous in design and comparison,^[13]^ we will conduct data synthesis using Review Manager Software 5.3. The fixed-effects or random-effects model will be chosen depending on the I^2^ value. A 95% confidence interval will be the effective size for data synthesis. We will perform qualitative analysis if the data is not fit for quantitative analysis.

#### 2.7.4 Subgroup analysis

We will perform subgroup analysis among patient conditions, treatment methods, and outcome measurements if feasible.

#### 2.7.5 Sensitivity analysis

We will perform sensitivity analysis to examine the robustness and reliability of merged outcome results with the exclusion of small and low-quality studies.

#### 2.7.6 Reporting bias

If enough trials (≥ 10 trials) are included, we will assess publication bias using funnels plots. Otherwise, we will perform the Egger test with STATA 13.0 Software.

#### 2.7.7 Grading the quality of evidence

We will use the Grading of Recommendations Assessment, Development, and Evaluation^[15]^ to assess the quality of evidence of the main outcomes, including the five aspects (study limitations, inconsistency, imprecision, indirectness, and publication bias). The quality of evidence will be graded as high, moderate, low, and very low.

#### 2.7.8 Ethics and dissemination

Given that this is a systematic review of the effectiveness of acupuncture and rehabilitation in patients with PSD, it does not involve individual experiments. Therefore, ethical approval will not be required. Upon completion of analyses, the results will be reported in a peer-reviewed journal.

## 3 Discussion

A systematic literature review by Hackett in 2005 reported that physical disability, stroke severity, and cognitive impairment were the most consistently associated with PSD.^[16]^ In 2014, Hackett et al performed an updated literature review and confirmed that physical disability in the acute and subsequent stroke phases, as well as stroke severity, are consistently associated with depression.^[17]^ Therefore, addressing physical disability may be crucial to the PSD treatment.

The intervention factors in this study will be acupuncture and rehabilitation. In ancient China, acupuncture therapy was used solely for stroke. However, acupuncture and rehabilitation training can currently be used to attenuate the neurological impairment degree and improve dysfunction.^[18]^ We speculate that this might be among the mechanisms underlying the treatment effects of acupuncture and rehabilitation training on PSD, that is, this treatment effects might involve the reduction of the physical disability degree.

To test this hypothesis, we will perform a systematic review and meta-analysis to estimate the efficacy of acupuncture combined with rehabilitation training in PSD treatment. Moreover, we will employ indices reflective of the nerve defect degree as secondary outcomes, including the National Institute of Health Stroke Scale scores and activities of daily living. We hope that this study will provide more evidence for clinical practitioners and health policy-makers in PSD treatment by analyzing and integrating published RCTs. However, this review may possess several limitations. First, our results might be affected by the quality of Chinese and English studies. Moreover, it is difficult to implement the double- or single-blind approach while assessing acupuncture interventions.

## Author contributions

**Conceptualization:** Yonghui Hou, Jieming Hao

**Data collection:** Yonghui Hou, Ying Liu

**Formal analysis:** Yonghui Hou, Ying Liu

**Funding acquisition:** Wenbin Fu, Ding Luo

**Software:** Yonghui Hou, Ying Liu

**Supervision:** Zehuai Wen, Baile Ning

**Drafting the manuscript:** Yonghui Hou, Xiangzhi Wang

**Reviewing & editing the manuscript:** Yonghui Hou, Ning Zhang
